# A two-cohort study on the association between the gut microbiota and bone density, microarchitecture, and strength

**DOI:** 10.3389/fendo.2023.1237727

**Published:** 2023-09-21

**Authors:** Paul C. Okoro, Eric S. Orwoll, Curtis Huttenhower, Xochitl Morgan, Thomas M. Kuntz, Lauren J. McIver, Alyssa B. Dufour, Mary L. Bouxsein, Lisa Langsetmo, Samaneh Farsijani, Deborah M. Kado, Roberto Pacifici, Shivani Sahni, Douglas P. Kiel

**Affiliations:** ^1^ Hinda and Arthur Marcus Institute for Aging Research, Hebrew SeniorLife, Boston, MA, United States; ^2^ Department of Medicine, Oregon Health & Sciences University, Portland, OR, United States; ^3^ Harvard Chan Microbiome in Public Health Center, Harvard T.H. Chan School of Public Health, Boston, MA, United States; ^4^ Department of Biostatistics, Harvard T.H. Chan School of Public Health, Boston, MA, United States; ^5^ Department of Immunology and Infectious Diseases, Harvard T.H. Chan School of Public Health, Boston, MA, United States; ^6^ Infectious Disease and Microbiome Program, Broad Institute of MIT and Harvard, Cambridge, MA, United States; ^7^ Department of Medicine, Beth Israel Deaconess Medical Center, Harvard Medical School, Boston, MA, United States; ^8^ Endocrine Unit, Massachusetts General Hospital, Harvard Medical School, Boston, MA, United States; ^9^ Department of Orthopedic Surgery, Harvard Medical School and Center for Advanced Orthopedic Studies, Beth Israel Deaconess Medical Center, Boston, MA, United States; ^10^ Center for Care Delivery and Outcomes Research, Minneapolis Veterans Affairs (VA) Health Care System, Minneapolis, MN, United States; ^11^ Department of Medicine, University of Minnesota, Minneapolis, MN, United States; ^12^ Department of Epidemiology, University of Pittsburgh, Pittsburgh, PA, United States; ^13^ Center for Aging and Population Health, University of Pittsburgh, Pittsburgh, PA, United States; ^14^ Department of Medicine, Stanford University, Stanford, CA, United States; ^15^ Geriatric Research Education and Clinical Center (GRECC), VA Health System, Palo Alto, CA, United States; ^16^ Division of Endocrinology, Metabolism and Lipids, Department of Medicine, Emory University School of Medicine, Atlanta, GA, United States

**Keywords:** gut microbiome, 16S amplicon sequencing, bone Density, bone microarchitecture, cohort study, aging

## Abstract

The gut microbiome affects the inflammatory environment through effects on T-cells, which influence the production of immune mediators and inflammatory cytokines that stimulate osteoclastogenesis and bone loss in mice. However, there are few large human studies of the gut microbiome and skeletal health. We investigated the association between the human gut microbiome and high resolution peripheral quantitative computed tomography (HR-pQCT) scans of the radius and tibia in two large cohorts; Framingham Heart Study (FHS [n=1227, age range: 32 – 89]), and the Osteoporosis in Men Study (MrOS [n=836, age range: 78 – 98]). Stool samples from study participants underwent amplification and sequencing of the V4 hypervariable region of the 16S rRNA gene. The resulting 16S rRNA sequencing data were processed separately for each cohort, with the DADA2 pipeline incorporated in the16S bioBakery workflow. Resulting amplicon sequence variants were assigned taxonomies using the SILVA reference database. Controlling for multiple covariates, we tested for associations between microbial taxa abundances and HR-pQCT measures using general linear models as implemented in microbiome multivariable association with linear model (MaAslin2). Abundance of 37 microbial genera in FHS, and 4 genera in MrOS, were associated with various skeletal measures (false discovery rate [FDR] ≤ 0.1) including the association of *DTU089* with bone measures, which was independently replicated in the two cohorts. A meta-analysis of the taxa-bone associations further revealed (FDR ≤ 0.25) that greater abundances of the genera; *Akkermansia* and *DTU089*, were associated with lower radius total vBMD, and tibia cortical vBMD respectively. Conversely, higher abundances of the genera; *Lachnospiraceae NK4A136 group*, and *Faecalibacterium* were associated with greater tibia cortical vBMD. We also investigated functional capabilities of microbial taxa by testing for associations between predicted (based on 16S rRNA amplicon sequence data) metabolic pathways abundance and bone phenotypes in each cohort. While there were no concordant functional associations observed in both cohorts, a meta-analysis revealed 8 pathways including the super-pathway of histidine, purine, and pyrimidine biosynthesis, associated with bone measures of the tibia cortical compartment. In conclusion, our findings suggest that there is a link between the gut microbiome and skeletal metabolism.

## 1 Introduction

The community of commensal microbes that reside in the gut may represent a potentially modifiable factor contributing to skeletal health. The gut microbiome has been shown to affect the inflammatory environment through effects on the T-cell landscape, which influence the production of soluble immune mediators and inflammatory cytokines that stimulate osteoclastogenesis and bone loss in mice (1–3). In fact, there are many potential biologic links between the gut microbiome and skeletal metabolism, such as effects on inflammation mediated by the production of short chain fatty acids (4), as well as metabolism of dietary components such as vitamin K (5), vitamin D (6), and complex polysaccharides (7). These mechanistic links between the gut microbiota and the skeleton have been reviewed recently in multiple publications and the term “osteomicrobiology” has been used to characterize this relationship (8). Despite the growing body of literature supporting a gut microbiota – bone connection, there are few large clinical investigations of this association.

We recently studied a sample of 831 older community dwelling men (age range: 78 – 98 years) from the Osteoporosis in Men Study (MrOS) whose stool specimens were collected and the 16S rRNA gene V4 hypervariable sequenced to determine if microbial abundances were associated with measures of bone density, microarchitecture and strength of the distal radius and tibia (9). Abundances of four bacterial genera (*Anaerofilum*, *Methanomassiliicoccus, Ruminiclostridium*, and *Tyzzerella*) were weakly associated with bone density, structure, or strength, and the measured directions of associations of genera were generally consistent across multiple bone measures, supporting a role for microbiota on skeletal homeostasis (9). These results suggested that although bone-microbiome associations can be observed, the magnitude of association is modest, and future studies would require larger sample sizes. In addition, given that the core microbiota of older study participants is reported to be distinct from that previously established for younger adults (10), we have now added another larger sample of largely middle-aged men and women to our previous report, to identify new taxa-bone associations, as well as being able to look for consistency across cohorts.

## 2 Methods

### 2.1 Description of study cohorts and participants

#### 2.1.1 The framingham heart study

The FHS began in 1948 with the recruitment of a community-based sample of residents from the town of Framingham, Massachusetts and surrounding towns to study the risk factors for heart disease. Over the subsequent decades, offspring of the original participants were recruited with their spouses into the Offspring Cohort, and finally the children of the Offspring and their spouses were recruited into the third-generation cohort (Gen3). Additional Offspring cohort spouses were also included, as they were evaluated along with the Gen3 participants. Finally, a multi-ethnic cohort, the Omni cohort, was recruited from the same towns as the Gen3 participants, and were evaluated along with the Gen3 participants. The methods used to collect gut microbiome samples from FHS Gen3, the additional spouses and the Omni cohort participants have been well described in a previous study including their sub-cohort information (11). In addition to stool collection, participants were queried about past colon surgery and use of antibiotics in the 30 days before stool collection, as these two criteria were used to exclude potential participants from our analysis. A total of 1424 stool samples were processed for 16S rRNA gene amplicon sequencing.

#### 2.1.2 The osteoporotic fractures in men study

The MrOS cohort is comprised of community-dwelling older men, recruited at six clinical sites in the U.S between 2000 and 2002. The cohort and recruitment methods have been previously described (12, 13), including a smaller study of the gut microbiome and skeletal phenotypes (9). In the MrOS cohort, 920 participants’ stool samples were processed for 16S rRNA gene amplicon sequencing. Questionnaire on antibiotics use within 30 days before stool collection was used to exclude samples before downstream analysis.

### 2.2 Stool sample collection, 16S rRNA gene sequencing, and sequence data preprocessing

#### 2.2.1 FHS

As previously described (11) stool samples were collected in 100% ethanol, and bacterial DNA extracted using a Qiagen custom protocol and stored at -20°C. The V4 hypervariable region of the 16S rRNA gene was targeted and amplified with the 515F primer (5′-AATGATACGGCGACCACCGAGATCTACACTATGGTAATTGTGTGCCAGCMGCCGCGGTAA-3′) and unique reverse barcode primers from the Golay primer set (14, 15). The amplified PCR products were sequenced on an Illumina MiSeq machine with the 2 x 150 base pair paired-end protocol. The average reads per sample was 77,509 (S.D = 27,957), ranging from 3,990 to 187,222.

#### 2.2.2 MrOS

As described previously (9, 16) 920 stool samples were received, and then processed and sequenced in two consecutive batches (first batch, n=600; second batch, n=320) (9). The fecal samples were collected at home by study participants using the Omnigene Gut collection kit, and stored at -80°C until DNA extraction. Bacterial DNA was extracted using the MO BIO Powersoil DNA Isolation Kit. The 16S rRNA gene V4 region was targeted and amplified by PCR using 515F and 806R primers. The resulting PCR products were sequenced on the Illumina MiSeq machine using the 2 x 250 base pair paired-end protocol. The average reads per sample was 42,310 (S.D = 13,404), ranging from 9,765 to 108,521.

### 2.3 Preprocessing of 16S rRNA samples in the FHS and MrOS

For each of the study cohorts, the resulting 16S rRNA gene amplicon sequencing data were separately processed with DADA2 (17) denoising pipeline incorporated in the 16S bioBakery workflow built with AnADAMA2 (18). Briefly, the paired-end sequence data were de-multiplexed, after which DADA2 denoising, filtering, trimming (FHS = 140 forward and 135 reverse, MrOS = 225 forward and 175 reverse), merging, chimera removal and taxonomic assignment were executed in R. This yielded total denoised ASVs of 36,737 in FHS, and 12,799 in MrOS. The resulting ASVs were assigned taxonomies using the SILVA reference database (19). Specifically, for genus level classification we used SILVA version 132, while version 128 was used for species level classification. For downstream analysis, we used Phyloseq (20) version 1.38.0 to generate two separate taxa count tables from the ASVs count tables; one glomed at genus levels, and another at species levels. Specifically, microbial taxa counts glomed at genus levels were used for microbial diversity analysis, as well as association testing with bone phenotypes, while taxa counts at a species level were used only for taxa-bone association tests. In the genus level count table, there was 391 genera in FHS, and 359 in MrOS. The species level count table had 275 species in FHS, and 273 in MrOS. For downstream association tests, the resulting microbial taxa count tables were converted to relative abundance. Because the MrOS 16S rRNA data were processed in two batches, we used MMUPHin (21) version 1.8.2 to adjust for batch effects in the taxa count table.

### 2.4 Metagenome prediction

We used PICRUSt2 software (22) to predict MetaCyc (23) pathways functional abundance from the 16S rRNA amplicon sequence data. This works in such a way that many amplicon sequence variants (ASVs) contribute in the functional features abundance predictions, and thus the ASVs matched to microbial taxa are not necessarily the ones we would find in taxa-bone associations as top most signal. There were 394 predicted pathways in FHS and 404 in MrOS cohorts. To reduce high dimensionality of our predicted functional features, and reduce redundancies in our tests, we removed features that were very highly correlated (Person Correlation > 0.9) with each other. To achieve this, we used “findCorrelation” function in the caret package in R. In case two features are highly correlated, the function considers the mean absolute correlation of each feature and removes the one with the largest mean absolute correlation. The resulting pathways count tables were further filtered to exclude functional features present in less than 10% of samples, and then converted to relative abundance. Specifically, for MrOS, we also adjusted for batch effects in the functional abundance table. The number of final functional features retained for downstream association tests were 112 in FHS, and 139 in MrOS.

### 2.5 Skeletal measurements

As previously described (9), high resolution peripheral quantitative computed tomography (HR-pQCT) was performed to measure bone density, microarchitecture and strength at the distal radius and tibia using Scanco XtremeCT II machines (Scanco Medical AG, Brüttisellen, Switzerland). Scans were obtained and processed similarly in the two cohorts, including careful quality control and standard image analysis with micro-finite element analysis μFEA (24–26). For the MrOS cohort, the radius scan started at a distal 4% site derived from manual limb measurement. The tibia scan started at a distal 7.3% of the tibial length. For the FHS, the non-dominant radius scan start site was set at 4% of the radius length after measuring arm length, and the right tibial scan start site was set at 7.3% of the tibia length. During quality control, we used a five-point movement artifact grading where a larger grade indicated worse movement artifact (27). Grade 4 scans were used for all density measures but not for micro-architectural measures like Trabecular number (Tb_N), Trabecular thickness (Tb_Th), Trabecular separation (Tb_Sp), Standard deviation of 1/Tb_N (Tb.1/N.SD1), cortical porosity (CtPo). Radial and tibial skeletal phenotypes included: 1) Volumetric BMD of the total (Tot_vBMD), trabecular (Tb_vBMD), inner trabecular (Tb_Inn_vBMD) and cortical regions (Ct_vBMD); 2) Cross-sectional area of the total (Ct_Area) and cortical bone area fraction, or the percentage of the total area occupied by the cortex (Ct_BATA); 3) Trabecular number and thickness; and 4) Cortical thickness (Ct_Th). Instead of using cortical porosity, we chose to test for associations with cortical BMD, which accounts for losses of density due to pores, since the assessment of cortical porosity is limited to moderately sized pores only. For both studies, the failure load was estimated using the method described by Pistoia et al., 2002 (28).

### 2.6 Covariate measurements

In this study, the clinical characteristics and covariates that we adjusted for include; age, sex, body mass index (BMI, kg/m^2^), smoking (yes/no), diabetes (yes/no), hospitalization in the past year (yes/no), total number of medications used (as count), use of proton pump inhibitors (PPI), and metformin, race (self-reported), Diet Quality Index (DQI) (26, 29, 30), and clinic site (in MrOS alone). Smoking status was based on self-report, and individuals were classified as current smokers vs not currently smoking (past smokers and those who had never smoked). Diabetes status was defined as current use of a medication indicated for the treatment of diabetes or having either a fasting blood glucose level of ≥126 mg/dL or a random blood glucose level of ≥200 mg/dL. Participants with normal blood glucose <126 mg/dL and taking no diabetes medications were considered as non-diabetic. Recognizing that a recent hospitalization might alter the gut microbiota, we classified participants as to whether they had a hospitalization in the past year or not. We also ascertained the use of several medications that might alter the gut microbiome; metformin and proton pump inhibitors, as well as total number of medications. Participants self-reported their race as one of four categories; African American, Asian, Hispanic, White or Other. We accounted for dietary factors by creating the DQI using food frequency questionnaires (FFQ). In the FHS Gen3/Omni2/New Offspring Spouse (NOS) cohorts, dietary assessment was conducted in 2008-2011 using the Willett 126-item semi-quantitative FFQ (31). The relative validity of this FFQ has been evaluated for both nutrients and foods in other studies (31–34). For participants with missing FFQ, we used their dietary information from the previous examination in 2002-2005. In the MrOS cohort, dietary assessment was conducted in 2014-2016 using the Block FFQ, a 69-item semi-quantitative FFQ. This FFQ was derived from the original validated Block 98 FFQ (35).

Dietary information from both cohorts was considered valid if energy intakes were ≥2.51 MJ/d (600 kcal/d) and <6.74 MJ/d (4,000 kcal/d) for women and <17.54 MJ/d (4,200 kcal/d) for men, and <13 food items were left blank. We assessed diet quality with the DQI Revised (DQI-R), a 10-component estimate of diet quality relative to national guidelines (26, 29, 30). The DQI-R incorporates the following dietary variables as estimated from the FFQs in FHS and MrOS: percent of energy intake from fat; percent of energy intake from saturated fat; dietary cholesterol; fruit servings; vegetable servings; grain servings; calcium intake; iron intake; dietary diversity; and dietary moderation. Each component can contribute up to 10 points to an overall diet quality score ranging from 0 (lowest quality) to 100 (highest quality). Since the number of food items available from the FFQ in FHS was higher than the FFQ in MrOS, we collapsed these additional food items in the FHS into a single food category that matched with the food category available in MrOS. This ensured appropriate data harmonization before calculation of DQI-R. Additionally, to prevent unhealthy foods from artificially driving up the dietary diversity score, we removed fruit juice and processed meats from sub-categories of fruit intake and protein intake, which were then used to calculate dietary diversity score.

### 2.7 Statistical analysis

Analyses were performed in R (v 4.1.2) using the following packages: Phyloseq (20) (v 1.38.0) for microbial diversity analysis, MaAsLin2 (36) (v 1.8.0) for multivariable association modeling, MMUPHin (21) (v 1.8.2) for batch corrections and meta-analysis, and Vegan (37) (v 2.6.2) for microbial PERMANOVA analysis.

#### 2.7.1 Analytical approach

Firstly, the covariates and HR-pQCT bone measures of the two cohorts were compared using Student’s T test or Chi Squared test and P values < 0.05 were considered significant. Next, we assessed and compared the abundance and beta diversity of the gut microbiome from the two cohorts. We carried out multivariable analyses separately in each of the two cohorts on the microbial taxa relative abundance profiles and bone phenotypes. Because the FHS cohort contained both male and female participants, we also performed sex specific analysis for the FHS cohort. To potentially investigate the effect of sample size and sex on the number of significant taxa-bone associations detected, we also tested for taxa-bone association using a randomly subsampled FHS cohort of 836 samples (male=384, female=452). We further combined the two cohorts by conducting a meta-analysis of male only participants (all of MrOS and males in FHS), and another meta-analysis of both male and female participants (all of FHS and MrOS). Finally, we also tested for association between predicted functional pathways and bone measures in the individual cohorts as well as meta-analysis of both cohorts’ pathway-bone associations, but did not conduct sex specific analysis, and FHS subsampling.

#### 2.7.2 Microbiome abundance and diversity:

We processed the 16S rRNA amplicon sequence data from the two cohorts separately using DADA2 (17). The resulting taxa count table were glomed at genus level using Phyloseq (20) and converted to relative abundance. The microbiome of the male and female participants in the FHS cohorts were compared using alpha diversity (Observed genera - richness, and Shannon), and beta diversity (Bray-Curtis, and weighted UNIFRAC distances), with covariates adjusted PERMANOVA test for sex differences calculated. Specifically, to estimate richness as measured by the observed genera, we first rarefied the FHS samples. We also estimated beta diversity using Bray-Curtis distance on the combined FHS-MrOS abundance table, with covariates adjusted PERMANOVA test for cohort differences calculated. All beta diversity estimates were visualized using Non-Metric Multidimensional Scaling (NMDS).

#### 2.7.3 Multivariable associations

We fit a general linear model as implemented in MaAslin2 (36) using its default parameters (min_abundance = 0.0, min_prevalence = 0.1, analysis_method=“LM”, normalization=“TSS”, transform=“LOG”, max_significance=0.25), including all the covariates, HR-pQCT bone measures, and microbial taxa abundances. Using the same parameters and controlling for covariates, we also analyzed the association of predicted MetaCyc (38) pathway abundances and HR-pQCT bone measures. MMUPHin (21) was used to conduct meta-analysis of the two study cohorts. MMUPHin works by first performing multivariable regression analysis with MaAslin2, and then aggregating the results with established fixed/mixed effect models. The associations’ P-values were corrected for multiple testing using Benjamini & Hochberg false discovery rate (FDR) control method. The association tests linear model with all covariates as conducted in MaAslin2 are captured in the formula below.

##### 2.7.3.1 FHS

*Genera Relative Abundance ~ HRPQCT + SEX + AGE + SMOKING + BMI + DIABETES + HOSPITALIZATION + TOTAL MEDCATIONS + PPI + METFORMIN + RACE + DQI-R*

*Species Relative Abundance ~ HRPQCT + SEX + AGE + SMOKING + BMI + DIABETES + HOSPITALIZATION + TOTAL MEDICATIONS + PPI + METFORMIN + RACE + DQI-R*

*Pathway Relative Abundance ~ HRPQCT + SEX + AGE + SMOKING + BMI + DIABETES + HOSPITALIZATION + TOTAL MEDICATIONS + PPI + METFORMIN + RACE + DQI-R*

##### 2.7.3.2 MrOS

*Batch Adjusted Genera Relative Abundance ~ HRPQCT + AGE + SMOKING + BMI + DIABETES + HOSPITALIZATION + TOTAL MEDICATIONS + PPI + METFORMIN + RACE + DQI-R | CLINIC SITE*

*Batch Adjusted Species Relative Abundance ~ HRPQCT + AGE + SMOKING + BMI + DIABETES + HOSPITALIZATION + TOTAL MEDICATIONS + PPI + METFORMIN + RACE + DQI-R | CLINIC SITE*

*Batch Adjusted Pathway Relative Abundance ~ HRPQCT + AGE + SMOKING + BMI + DIABETES + HOSPITALIZATION + TOTAL MEDICATIONS + PPI + METFORMIN + RACE + DQI-R | CLINIC SITE*

## 3 Results

### 3.1 Characteristics of study populations and participants

**Figure 1:**
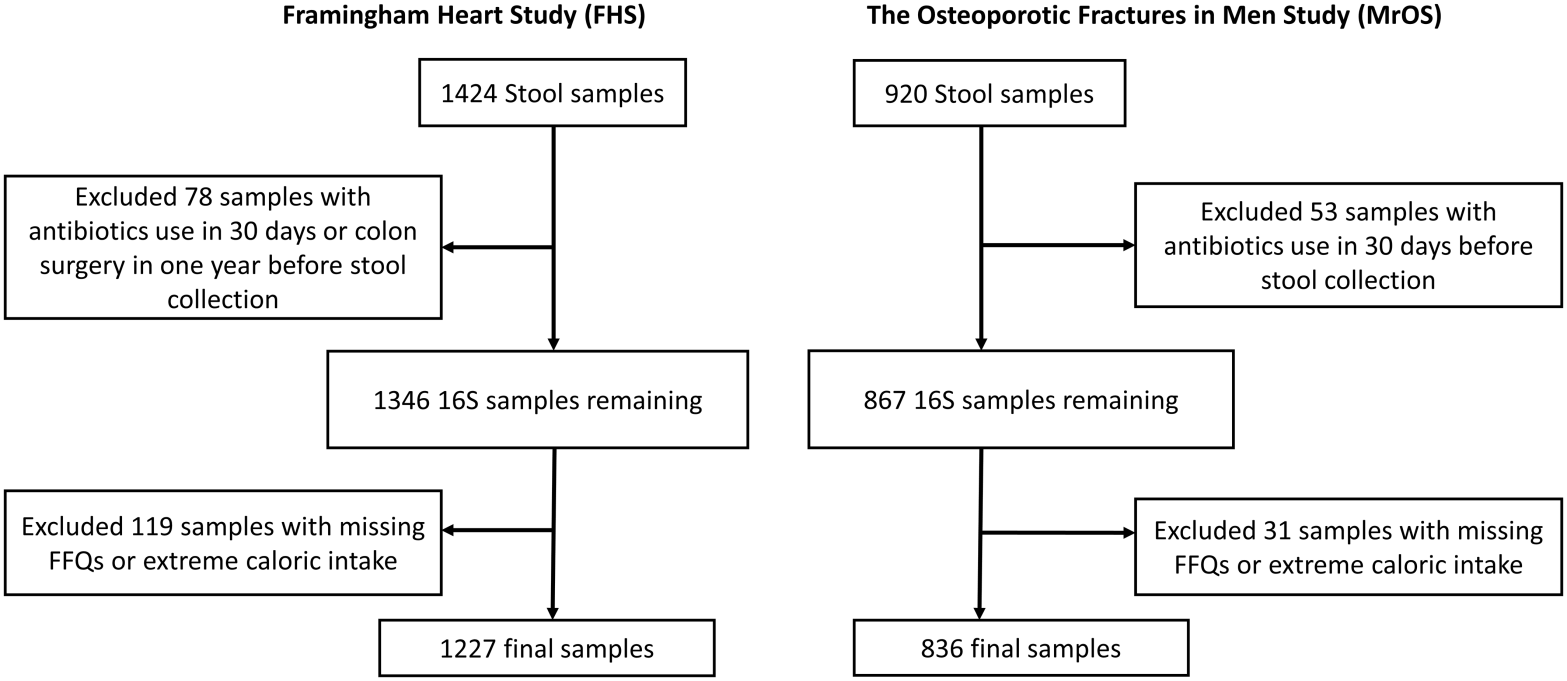
Flowchart of stool samples processed for microbial profiling using 16S ribosomal RNA (rRNA) sequencing. Identical criteria were applied to exclusions of samples from the Framingham Heart Study and the Osteoporotic Fractures in Men Study cohorts including participants who reported use of antibiotics within 30 days of collection, a history of colon surgery in the year prior to collection, and samples from individuals lacking covariate data.

Our study included participants from two large observational studies; the Framingham Heart Study (FHS) Generation 3 cohort, and the Osteoporotic Fractures in Men (MrOS) Study. Stool samples received from each participant (FHS=1424, MrOS=920) were processed for 16S rRNA amplicon sequencing. Participants who had used antibiotics within last 30 days or had colon surgery in the year before stool collection (n=78 & 53, for FHS and MrOS respectively) were excluded from analysis (**Figure 1**). We also excluded participants with missing covariate data (n=119 & 31 with missing FFQs or extreme caloric intake, for FHS and MrOS respectively), resulting in a final analytic sample size of 1227 and 836 for FHS and MrOS respectively.

The demographics and clinical characteristics, and the HR-pQCT skeletal measures of the study participants are summarized in **Tables 1A**, **B**. Notably, the MrOS participants were all male, much older with an average age of 84.2 years (range; 78 – 98 years), and used an average of 9.1 medications (SD: 5.6). In contrast, the FHS participants were 44.3% male, with an average age of 55.2 years (range; 32 – 89 years), and used fewer medications (average 2.7 medications [SD: 3.0]). In both cohorts, the majority of participants self-reported race as “white” (FHS=91.2%, MrOS=89.5%). The two cohorts significantly differed (p<0.05) across all measures except for use of metformin, and radius cortical thickness. In addition, comparison of the FHS Men subgroup and MrOS showed that there were no significant difference in the use of metformin, and race, and HR-pQCT scans of radius total area, and tibia trabecular vBMD and trabecular inner vBMD. In the comparison of FHS Men and FHS Women, there were no statistical difference in age, smoking, hospitalization, use of proton pump inhibitors, and race, as well as HR-pQCT scan of radius cortical vBMD. It is important to note that the multiple HR-pQCT measurements of bone density, architecture and strength are correlated with each other as shown in **Supplementary Figure 1**. In general, HR-pQCT measures of the same bone compartment (cortical or trabecular) were moderately correlated (r>0.3). Measures of total bone area were moderately inversely correlated with most cortical and total vBMD measures.

**Table 1A:** Study sample characteristics.

Sample Characteristics of the Two Cohorts				
	FHS (N=1227)	FHS Women (N=683)	FHS Men (N=544)	MrOS (N=836)
Male, n (%)	544 (44.3)	0 (0)	544 (100)	836 (100)
Age, years (S.D)	55.2 (9.1)	54.8 (8.9)	55.6 (9.2)	84.2 (4.0)
Smoking, n (%)	77 (6.3)	41 (6.0)	36 (6.6)	10 (1.2)
BMI, Kg/m2 (S.D)	28.0 (5.5)	27.2 (5.9)	29.0 (4.8)	27.0 (3.8)
Diabetes, n (%)	107 (8.7)	42 (6.1)	65 (11.9)	133 (15.9)
Recent Hospitalization, n (%)	85 (6.9)	51 (7.5)	34 (6.3)	168 (20.1)
Use of Proton Pump Inhibitor, n (%)	146 (11.9)	79 (11.6)	67 (12.3)	184 (22.0)
Use of Metformin, n (%)	80 (6.5)	35 (5.1)	45 (8.3)	59 (7.1)
Total number of Medications (S.D)	2.7 (3.0)	2.9 (3.2)	2.3 (2.8)	9.1 (5.6)
Revised Diet Quality Index (S.D)	74.2 (9.7)	75.3 (9.7)	72.7 (9.6)	50.1 (13.6)
Race, n (%)				
African American	16 (1.3)	8 (1.2)	8 (1.5)	27 (3.2)
Asian	34 (2.8)	18 (2.6)	16 (2.9)	30 (3.6)
Hispanic	21 (1.7)	10 (1.5)	11 (2.0)	18 (2.2)
White	1119 (91.2)	620 (90.8)	499 (91.7)	748 (89.5)
Other	37 (3.0)	27 (4.0)	10 (1.8)	13 (1.6)

The FHS cohort participants is comprised of both men and women as broken down in the table, while the MrOS cohort participants is comprised of only men. All sample characteristics differed between the two cohorts (p<0.05) except for the use of metformin. In the comparison of FHS Men and FHS Women, there were no statistical difference in age, smoking, hospitalization, use of proton pump inhibitors, and race. The comparison of FHS Men and MrOS showed that there were no significant difference in the use of metformin, and race.

**Table 1B:** Measures of skeletal phenotypes obtained using High Resolution Peripheral Quantitative Computed Tomography HR-pQCT scans.

Skeletal Phenotypes Measured using HR-pQCT Scans				
Phenotype	FHS (N=1227)	FHS Women (N=683)	FHS Men (N=544)	MrOS (N=836)
	Mean (SD)	Mean (SD)	Mean (SD)	Mean (SD)
Radius Bone Measures				
r_Ct_Th	0.96 (0.21)	0.86 (0.17)	1.09 (0.19)	0.96 (0.22)
r_Ct_vBMD	873.3 (65.3)	876.3 (73.2)	869.6 (53.9)	799.1 (66.3)
r_CtBATA	0.19 (0.05)	0.19 (0.04)	0.20 (0.04)	0.17 (0.05)
r_Tb_Inn_vBMD	123.4 (40.4)	103.3 (33.0)	148.0 (34.7)	133.4 (41.1)
r_Tb_N	1.51 (0.24)	1.41 (0.22)	1.64 (0.20)	1.40 (0.21)
r_Tb_vBMD	161.6 (41.0)	141.0 (33.8)	186.8 (34.3)	170.2 (39.4)
r_Tot_vBMD	296.0 (61.2)	278.8 (57.6)	321.8 (55.3)	275.6 (60.2)
r_Total_Area	332.5 (75.9)	282.2 (42.5)	394.8 (61.3)	398.3 (64.9)
r_fail_load_FEA	2926.2 (1072.6)	2217.6 (573.5)	3815.2 (872.1)	4921.7 (1339.9)
Tibia Bone Measures				
t_Ct_Th	1.62 (0.34)	1.44 (0.25)	1.83 (0.32)	1.49 (0.33)
t_Ct_vBMD	889.9 (76.0)	880.2 (88.1)	902.2 (54.8)	784.5 (78.1)
t_CtBATA	0.20 (0.04)	0.19 (0.04)	0.21 (0.04)	0.16 (0.04)
t_Tb_Inn_vBMD	121.2 (39.6)	108.0 (36.2)	137.8 (37.3)	135.3 (40.4)
t_Tb_N	1.39 (0.22)	1.35 (0.22)	1.44 (0.21)	1.34 (0.22)
t_Tb_vBMD	169.1 (41.5)	154.3 (37.6)	187.9 (38.4)	185.3 (38.5)
t_Tot_vBMD	309.2 (58.8)	287.6 (54.7)	336.3 (52.3)	281.9 (55.3)
t_Total_Area	744.0 (132.9)	670.1 (89.7)	836.9 (119.5)	893.5 (134.6)
t_fail_load_FEA	8468.6 (2355.6)	6880.5 (1321.3)	10466.1 (1780.0)	13691.8 (3009.8)

The FHS cohort participants is comprised of both men and women as broken down in the table, while the MrOS cohort participants is comprised of only men. All bone measures significantly differed between the two cohorts (p<0.05) except for radius cortical thickness. In the comparison of FHS Men and FHS Women, there was no statistical difference in HR-pQCT scan of radius cortical vBMD. The comparison of FHS Men and MrOS showed that there were no significant difference in the HR-pQCT scans of radius total area, and tibia trabecular vBMD and trabecular inner vBMD. The bone phenotypes were grouped based on bone site; radius (r) and tibia (t). Thus, bone variable names were prefixed with “r_” for radius bone measures, and “t_” for tibia bone measures. The bone phenotype labels are; Ct_Th, Cortical thickness (mm); Ct_vBMD, Average cortical vBMD (mg HA/cm3); CtBATA, Cortical bone area fraction, CtAr/TtAr; Tb_Inn_vBMD, Inner trabecular vBMD (mg HA/cm3); Tb_N, Trabecular number (1/mm); Tb_vBMD, Trabecular vBMD (mg HA/cm3); Tot_vBMD, Average total vBMD (mg HA/cm3); Total_Area, Total cross-sectional area (mm2); fail_load_FEA, Failure load, N.

#### 3.1.1 Microbial diversity of gut microbiota in study participants

**Figure 2:**
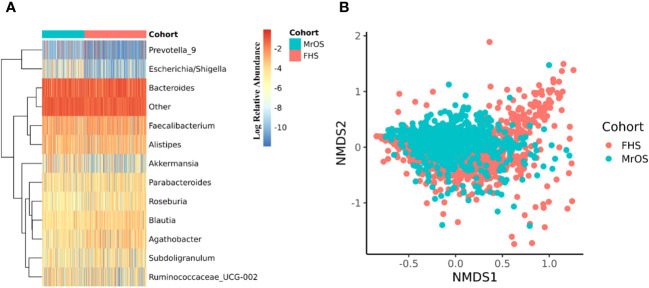
Microbiome diversity of the two cohorts. Both cohorts were separately evaluated for microbial composition and diversity. In each cohort, taxa abundances were glomed (merged) at the genus level. **(A)** shows a heatmap of the log relative abundance of the top ten most abundant genera within each cohort. In order to avoid 0 in the count table downstream during log transformation, 1 was uniformly added to the abundance table, and the abundance table was then converted to relative abundance within each cohort. Eight of the ten most abundant genera are the same in both cohorts. The group designated as "Other" includes all the remaining genera. For **(B)**, non-metric multidimensional scaling (NMDS) was used to ordinate the data, using Bray-Curtis distance as the beta diversity metric (Stress = 0.21). Cohort explained 3.0% of the total variability in the microbial abundance profiles (PERMANOVA, R2 = 0.030, P=0.001). We adjusted for all covariates in the PERMANOVA test.

The 16S rRNA amplicon sequence data of both cohorts were separately processed using DADA2 (17) incorporated in the 16S bioBakery workflow (18). Eight of the top 10 most abundant genera were the same across the two cohorts (**Figure 2A**). Two of the genera; *Akkermansia* and *Escherichia/Shigella*, that were in the top ten abundances for MrOS, were rarer in FHS, while *Agathobacter* and *Roseburia* were in the top ten for FHS but rarer in MrOS. We measured beta diversity using Bray-Curtis distance, and used Non-Metric Multidimensional Scaling (NMDS) to visualize the two cohorts (**Figure 2B**). While controlling for covariates, the total variability in the microbiota profiles due to study cohort differences was 3% (PERMANOVA test, P=0.001, R^2 = ^0.030). Relative to the total variability of microbial abundance, the contribution of being in one of the two cohorts was relatively small but significant. The cohort contribution to the microbiome variability may be attributed to several differences in abundances (**Figure 2A**) such as *Escherichia/Shigella* and *Akkermansia*, which were relatively rare in the FHS samples compared to the MrOS samples. Thus, microbiomes of the two cohorts are generally similar, but there are small but potentially important differences.

Next, because the FHS cohort is comprised of both male and female study participants, we further interrogated the sex differences in the FHS cohort by comparing the alpha and beta diversity of the two sex groups. We found no significant difference between males and females in their microbial complexity (Shannon index) and richness (Observed genera), as well as the beta diversity visualized with NMDS (**Supplementary Figure 2**). In fact, based on covariates adjusted PERMANOVA tests of Bray-Curtis (R^2^ = 0.005, P=0.001) and weighted UNIFRAC (R^2^ = 0.009, P=0.001) distances, sex only explained a very small fraction of total variability in the microbial abundance profiles of the FHS cohort.

#### 3.1.2 Within cohort associations between microbes and skeletal measures

Separately in each of the two study cohorts, we interrogated the association between the individual bone HR-pQCT measures and all the assayed microbial genera. We conducted cohort specific multivariable linear association models adjusting for covariates as implemented in MaAslin2 (36). Because to some extent this investigation was considered a hypothesis generating analysis, and because we also wanted to look for general patterns of association, we report findings at a less strict FDR ≤ 0.1.

#### 3.1.3 Associations between abundance of microbial genera and skeletal measures in the FHS

**Figure 3:**
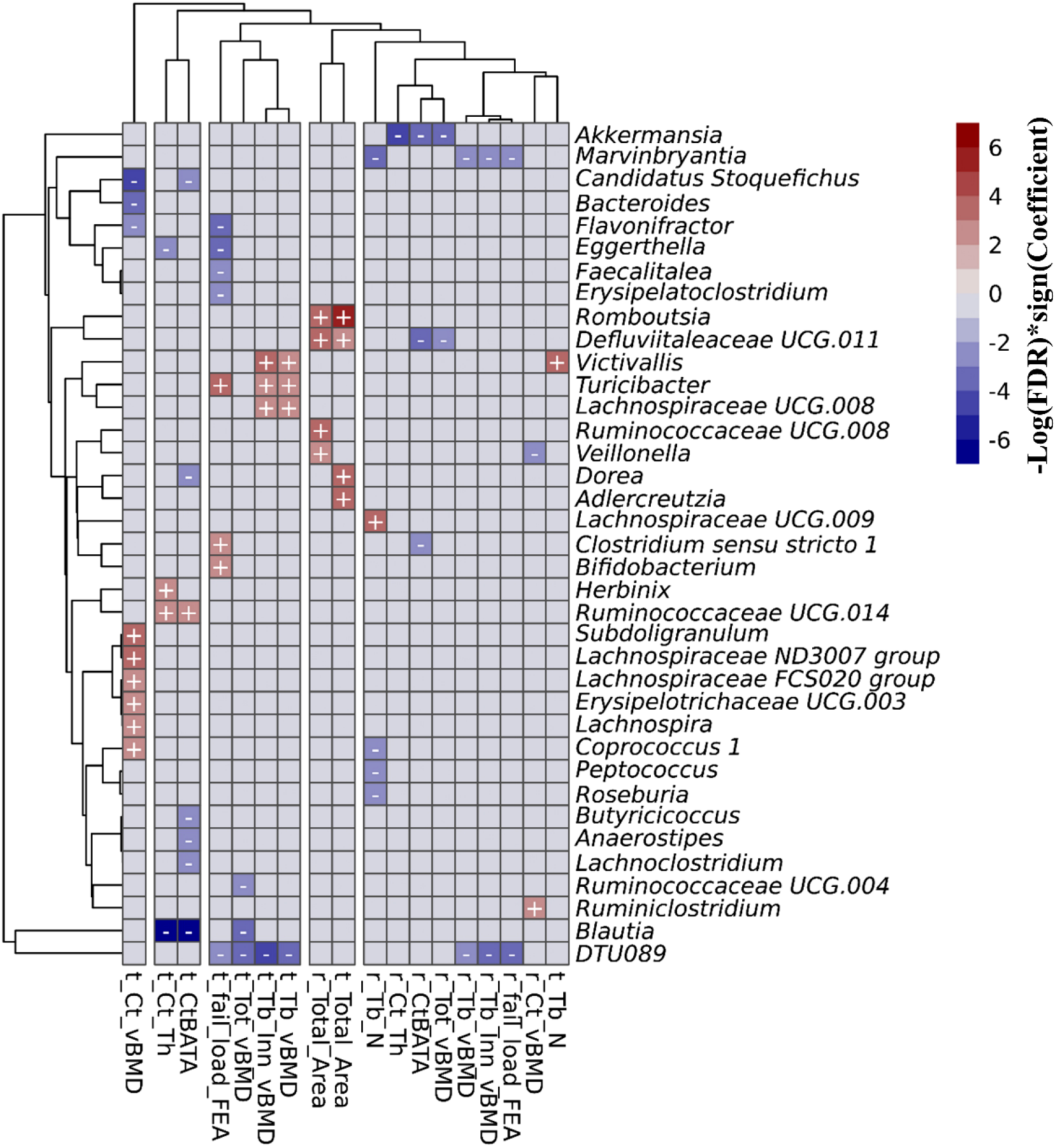
Associations between the gut microbial genera and bone measures in the FHS cohort. This includes all of the 67 associations with an FDR<0.1 that were calculated using MaAsLin2. We adjusted for all covariates in the association models. The X and Y axes were clustered hierarchically. The "+" and "-" signs indicate the direction of association between microbial abundance on the vertical axis and bone measures along the horizontal axis.

In the FHS cohort, we observed 67 taxa-bone associations (38 negative, and 29 positive) involving 37 distinct genera and the 18 HR-pQCT bone measures (**Figure 3**; **Supplementary Figure 3**). Taxa-bone associations with cortical bone measures represented 39% of the total, 27% involved the trabecular compartment, 19% involved total bone measures, and bone strength measures involved 15%. Interestingly, at both the radius and tibia bone sites, all microbial associations with total area measures were positive, indicating that greater abundance was associated with larger bone area, while associations with total vBMD were negative. A group of four genera were negatively associated with more than two bone measures*; DTU089, Marvinbryantia, Blautia*, and *Akkermansia*. Only *Turicibacter* and *Victivallis* had consistent positive associations with bone measures. We also observed that the genus *Defluviitaleaceae UCG.011* exhibited both positive and negative associations with bone measures, where abundance was positively associated with radius and tibial bone area but negatively associated with total vBMD and the percent of the radius that was cortical. Although not all associations were observed for both the tibia and radius, the direction of association was similar across bone sites. **Table 2** shows the strongest associations based on FDR ≤ 0.05, grouped into the four categories of skeletal bone measurements (cortical bone, trabecular bone, total bone, and bone strength measured by failure load), along with their individual test statistics. Specifically, across bone compartments at the radius, greater abundance of *Akkermansia* was associated with lower cortical thickness, ratio of cortical bone area to total area, and total vBMD. At the tibia bone site, increased abundance of *Blautia* was associated with lower cortical thickness, ratio of cortical bone area to total area, and total vBMD. At the radius and tibia, and across bone compartments, greater abundance of *DTU089* was associated with lower radius and tibia inner trabecular vBMD, tibia trabecular vBMD and total vBMD, and radius bone strength. Other associations between genera and bone were at either radius or tibia and in a single bone compartment. In general, we did not observe any specific pattern of associations that were unique to cortical or trabecular compartments or found only in a single skeletal site (radius vs tibia) (**Supplementary Figures 4**–**6**). Similar to a previous report from the MrOS cohort (9), we observed a greater number of significant associations for the weight bearing tibia, especially within the cortical bone compartment and for tibial bone strength (failure load). In general, the directions of associations were consistent across skeletal measures for individual organisms, and there were instances in which associations between microbes and density measures were opposite in direction than associations with skeletal area measures (**Supplementary Figure 5**).

**Table 2:** Associations between microbial genera abundance and bone measures in the FHS cohorts.

ALL FHS Cohort Microbial Genera – Bone Associations, FDR=0.05					
Genera	Phenotype	Beta	Stderr	P-val	Q-val
Cortical Bone Measures					
*Akkermansia*	r_Ct_Th	-0.586	0.169	5.69E-04	1.45E-02
*Blautia*	t_Ct_Th	-0.266	0.060	1.17E-05	7.47E-04
*Candidatus_Stoquefichus*	t_Ct_vBMD	-0.313	0.091	5.89E-04	1.20E-02
*Subdoligranulum*	t_Ct_vBMD	0.431	0.133	1.19E-03	2.01E-02
*Lachnospiraceae_ND3007_group*	t_Ct_vBMD	0.362	0.115	1.62E-03	2.43E-02
*Bacteroides*	t_Ct_vBMD	-0.131	0.046	4.30E-03	4.71E-02
*Akkermansia*	r_CtBATA	-0.472	0.145	1.13E-03	2.35E-02
*Defluviitaleaceae_UCG.011*	r_CtBATA	-0.239	0.08	2.74E-03	4.36E-02
*Blautia*	t_CtBATA	-0.215	0.052	3.66E-05	1.46E-03
Trabecular Bone Measures					
*DTU089*	r_Tb_Inn_vBMD	-0.373	0.127	3.38E-03	4.92E-02
*DTU089*	t_Tb_Inn_vBMD	-0.386	0.109	3.91E-04	9.24E-03
*Victivallis*	t_Tb_Inn_vBMD	0.300	0.097	2.10E-03	3.00E-02
*DTU089*	t_Tb_vBMD	-0.341	0.111	2.14E-03	3.11E-02
*Victivallis*	t_Tb_N	0.294	0.096	2.12E-03	2.87E-02
*Lachnospiraceae_UCG.009*	r_Tb_N	0.238	0.072	9.44E-04	2.30E-02
*Marvinbryantia*	r_Tb_N	-0.448	0.139	1.29E-03	2.71E-02
Total Bone Measures					
*Akkermansia*	r_Tot_vBMD	-0.456	0.147	1.96E-03	2.95E-02
*DTU089*	t_Tot_vBMD	-0.381	0.116	1.00E-03	1.87E-02
*Blautia*	t_Tot_vBMD	-0.179	0.056	1.28E-03	2.16E-02
*Romboutsia*	r_Total_Area	0.620	0.184	7.82E-04	2.27E-02
*Defluviitaleaceae_UCG.011*	r_Total_Area	0.381	0.115	9.88E-04	2.60E-02
*Ruminococcaceae_UCG.008*	r_Total_Area	0.328	0.102	1.39E-03	3.15E-02
*Romboutsia*	t_Total_Area	0.536	0.143	1.79E-04	5.94E-03
*Dorea*	t_Total_Area	0.344	0.120	4.23E-03	4.93E-02
*Adlercreutzia*	t_Total_Area	0.378	0.132	4.22E-03	4.93E-02
Bone Strength Measures					
*DTU089*	r_fail_load_FEA	-0.543	0.172	1.60E-03	3.90E-02
*Flavonifractor*	t_fail_load_FEA	-0.368	0.122	2.70E-03	4.57E-02
*Eggerthella*	t_fail_load_FEA	-0.432	0.144	2.83E-03	4.59E-02
*Turicibacter*	t_fail_load_FEA	0.496	0.166	2.85E-03	4.59E-02

Bone measures were collected using high resolution peripheral quantitative computed tomography. Taxa-bone associations with Benjamini & Hochberg false discovery rate (FDR) adjusted P-value (Q-value) ≤ 0.05 are shown. Skeletal phenotypes are grouped according to whether they were measured in cortical bone, trabecular bone, whole bone, or overall measures of skeletal strength. All associations were adjusted for covariates.

#### 3.1.4 Associations between abundance of microbial genera and skeletal measures in the MrOS

In contrast to the multiple associations detected in the FHS cohort, we did not observe microbial genera – bone association at an FDR ≤ 0.05 in the MrOS cohort. However, at a less strict FDR ≤ 0.1, we did observe five taxa-bone associations in the MrOS cohort at the tibia bone sites (**Table 3**). We found that increased abundance of the genera; *Methanobrevibacter and DTU089*, were associated with lower cortical vBMD, while increased abundance of *Lachnospiraceae NK4A136 group* was associated with greater cortical vBMD. Greater abundance of *Cloacibacillus* was associated with both greater trabecular inner vBMD and trabecular vBMD. Interestingly, as was observed in the FHS cohort, the negative association of *DTU089* with trabecular bone measures was consistent in the MrOS cohort for tibia cortical vBMD.

**Table 3:** Associations between microbial genera abundance and bone measures in the MrOS cohorts.

MrOS Cohort Microbial Genera – Bone Associations, FDR=0.1					
Genera	Phenotype	Beta	Stderr	P-val	Q-val
Cortical Bone Measures					
*Methanobrevibacter*	t_Ct_vBMD	-0.304	0.097	1.70E-03	5.44E-02
*DTU089*	t_Ct_vBMD	-0.211	0.070	2.49E-03	6.84E-02
*Lachnospiraceae NK4A136 group*	t_Ct_vBMD	0.246	0.081	2.61E-03	6.87E-02
Trabecular Bone Measures					
*Cloacibacillus*	t_Tb_Inn_vBMD	0.201	0.069	3.85E-03	9.64E-02
*Cloacibacillus*	t_Tb_vBMD	0.202	0.069	3.66E-03	9.14E-02

Bone measures were collected using high resolution peripheral quantitative computed tomography. Taxa-bone associations with Benjamini & Hochberg false discovery rate (FDR) adjusted P-value (Q-value) ≤0.1 are shown because there were no associations with Q-value ≤ 0.05. Skeletal phenotypes are grouped according to whether they were measured in cortical bone, trabecular bone, or whole bone, or overall measures of skeletal strength. All associations were adjusted for covariates.

#### 3.1.5 Associations between abundance of microbial species and skeletal measures in FHS and MrOS

**Figure 4:**
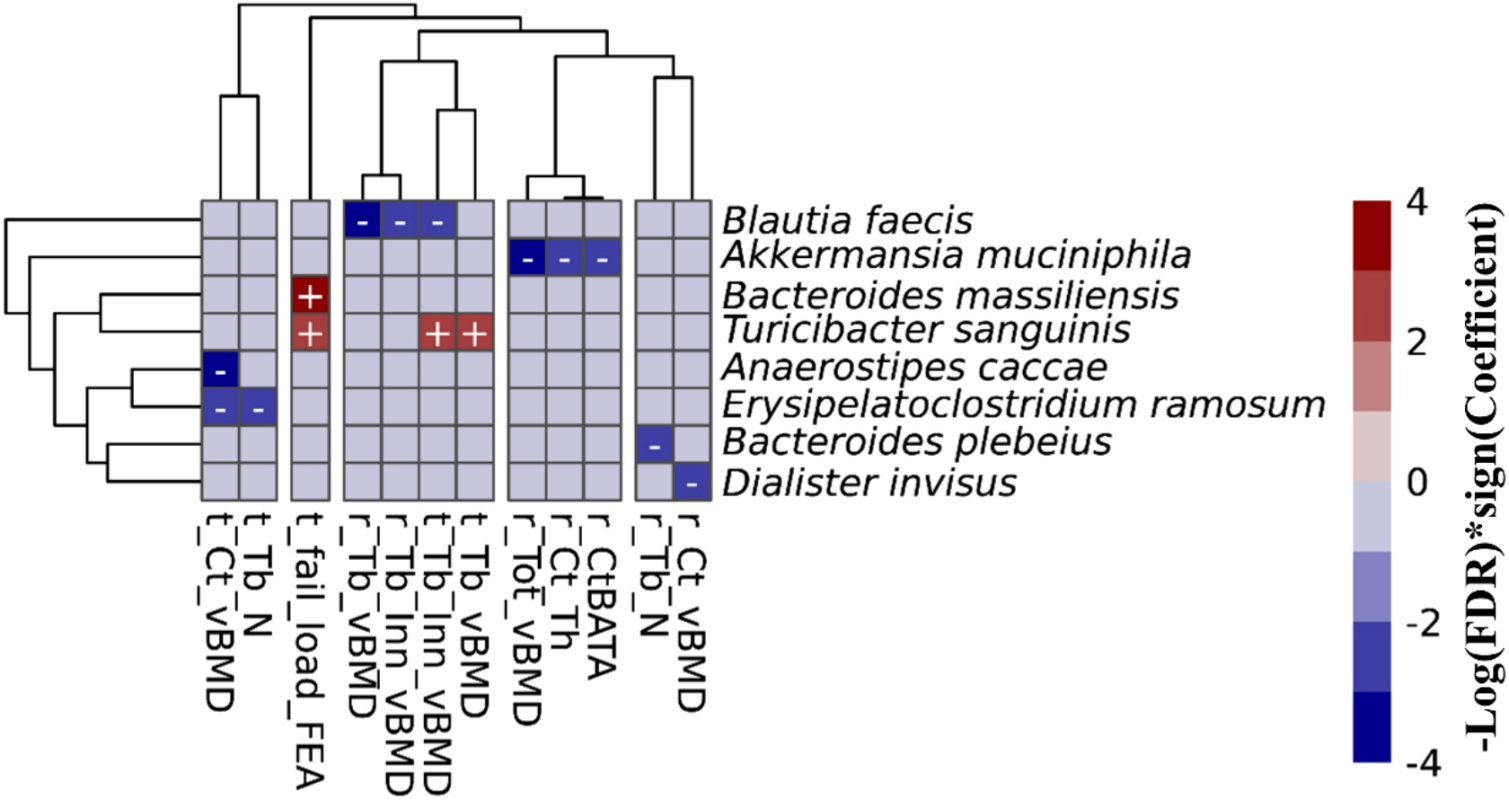
Associations between the gut microbial species and bone measures in the FHS cohort. This includes all of the 15 associations with an FDR ≤ 0.1 that were calculated using MaAsLin2. We adjusted for all covariates in the association models. The X and Y axes were clustered hierarchically. The “+” and “-“ signs indicate the direction of association between microbial abundance on the vertical axis and bone measures along the horizontal axis.

To refine the associations that we observed at the genus levels above, we further tested for separate associations (FDR value ≤ 0.10) between the abundance of microbial taxa at species levels (when species levels were able to be determined) and HR-pQCT bone measures. In the FHS cohort, we observed 15 taxa-bone associations (11 negative, and 4 positive) including 8 microbial species and 12 bone measures (**Figure 4**). Importantly, we observed that species were associated largely with the same bone measures and in the same direction of association, as was observed at the genus level association. In the MrOS cohort, we found only a beneficial association between *Lachnospira pectinoschiza* and radius cortical vBMD.

#### 3.1.6 Associations between abundance of microbial genera and skeletal measures in male, and female specific FHS, and subsampled FHS

We next sought to determine if the larger number of associations observed in FHS compared to MrOS was based on the MrOS cohort being comprised of only men in contrast to the FHS cohort that included both men and women. Thus, we computed FHS cohort sex-specific taxa-bone associations while controlling for the other covariates. The number of taxa-associations observed in either of the sex-specific analyses were relatively few, unlike the cohort specific results in the FHS cohort comprising both sexes. Specifically, the FHS male-only analysis (n=544) had 17 taxa-bone associations involving 7 distinct genera and 8 bone measures and the FHS female-only analysis had 17 taxa-bone associations between 9 genera and 11 bone measures (**Supplementary Figure 4**). To further probe the reduction in number of associations detected, we randomly subsampled the FHS cohort (male=384, female=452) to a sample size of 836, similar to the MrOS sample size, and tested for associations between microbial genera and bone measures. There were 33 taxa-bone associations involving 18 genera and 14 bone measures (**Supplementary Figure 4**). Thus, while the reasoning maybe more complex, the higher number of significant associations detected in the FHS cohort analysis, compared to the fewer associations in MrOS, may be due to sex differences and different sample sizes.

We provide a more comprehensive set of results, in **Supplementary Figures 5**, **6**, and **Supplementary Tables 1**-**9** including all the results for the cohort, sex specific, and subsampled-FHS taxa-bone associations up to FDR ≤ 0.25.

#### 3.1.7 Meta-analysis of the microbial abundance and skeletal measures’ associations in the two cohorts

To leverage the power of the two cohorts, we combined their taxa-bone associations in order to identify consistent overall effects, by conducting meta-analysis as implemented in the MMUPHin package (21). Due to the cohort differences as seen in the cohort specific analyses, and to accommodate further exploration, we report meta-analytic associations at a liberal FDR ≤ 0.25.

**Figure 5:**
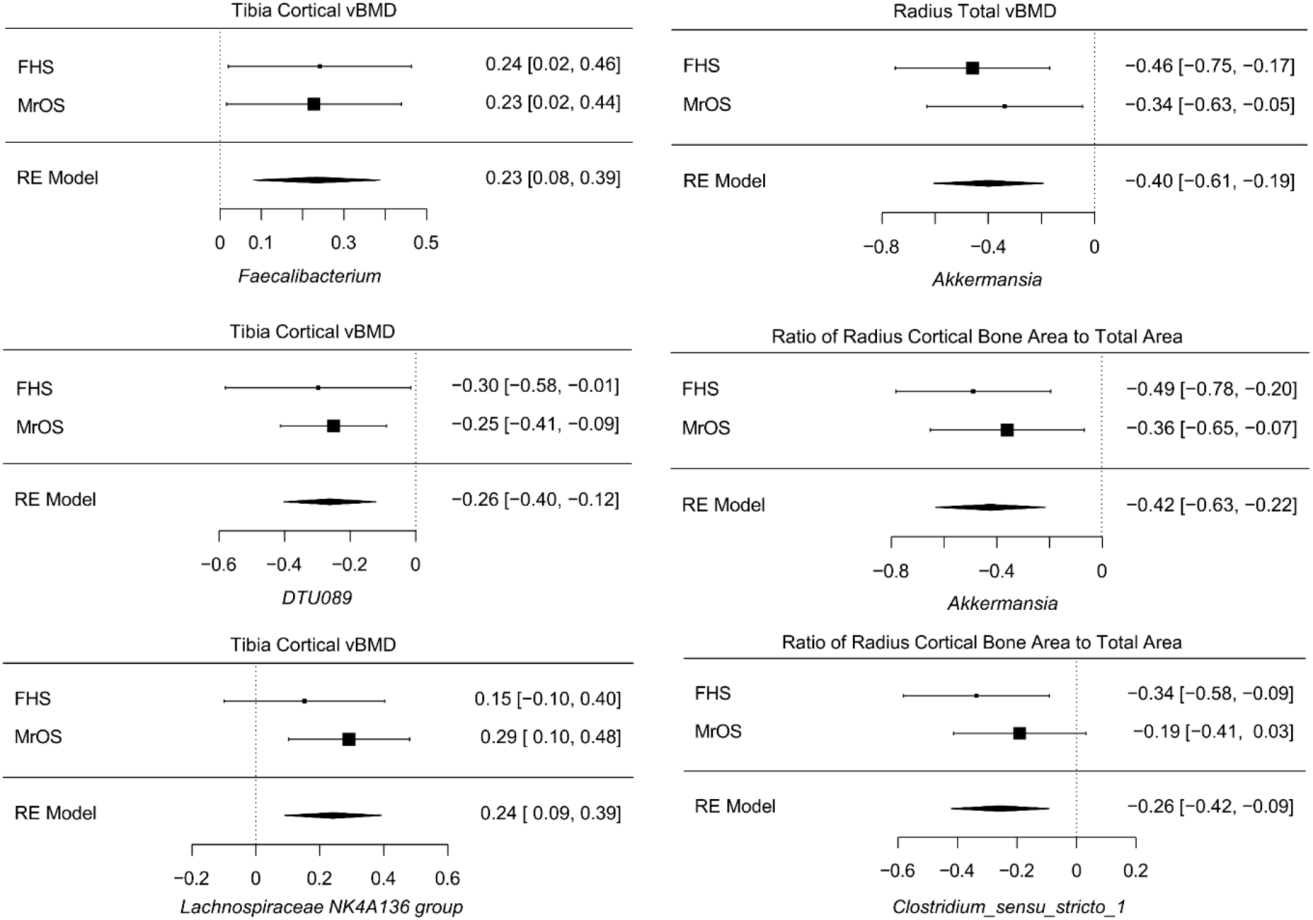
Meta-analysis of the associations between abundance of microbial genera and bone measures. Meta-analysis of genera - bone associations was conducted with MMUPHin and all associations at FDR ≤0.25 are shown.

Despite cohort differences, we found organisms that were consistently associated with bone phenotypes in both cohorts. At the genus level, the meta-analysis of all FHS and MrOS, resulted in 6 significant taxa-bone associations (**Figure 5**). Greater abundance of *Akkermansia* and *Clostridium_sensu_stricto_1* were both associated with lower ratio of radius cortical bone area to total area, and abundance of *Akkermansia* was associated with worse radius total vBMD. We also found that increased abundance of the genera; *DTU089*, *Lachnospiraceae NK4A136 group*, and *Faecalibacterium* were associated with lower tibia cortical vBMD. Interestingly, the inverse associations of *DTU089* and *Akkermansia* with tibia cortical vBMD, and radius total vBMD respectively, were equally observed in the meta-analysis of the FHS Male subgroup and MrOS. Importantly, these meta-analytic associations are consistent with the cohort specific analysis results.

The meta-analysis of the species level associations from the FHS and MrOS cohorts further confirmed the associations using genera, such as the direct association between abundance of *Akkermansia muciniphila* and measures at the radius such as the ratio of cortical bone area to total area at the radius, total vBMD, and cortical thickness. Other analyses of species level associations indicated that greater abundance of *Erysipelatoclostridium ramosum* was associated with lower tibia cortical vBMD, and that greater abundance of *Flavonifractor plautii* was associated with greater radius trabecular number. **Supplementary Tables 10**-**13** contain all meta-analysis summary statistics at FDR ≤ 0.25 for all FHS and MrOS meta-analysis, as well as FHS male group and MrOS meta-analysis respectively.

#### 3.1.8 Associations between abundance of predicted functional pathway and skeletal measures

We explored the associations between bone measures and abundance of predicted metabolic pathways (See Methods). After multivariable adjustment, we identified several pathways associated with multiple bone sites and compartments.

**Figure 6:**
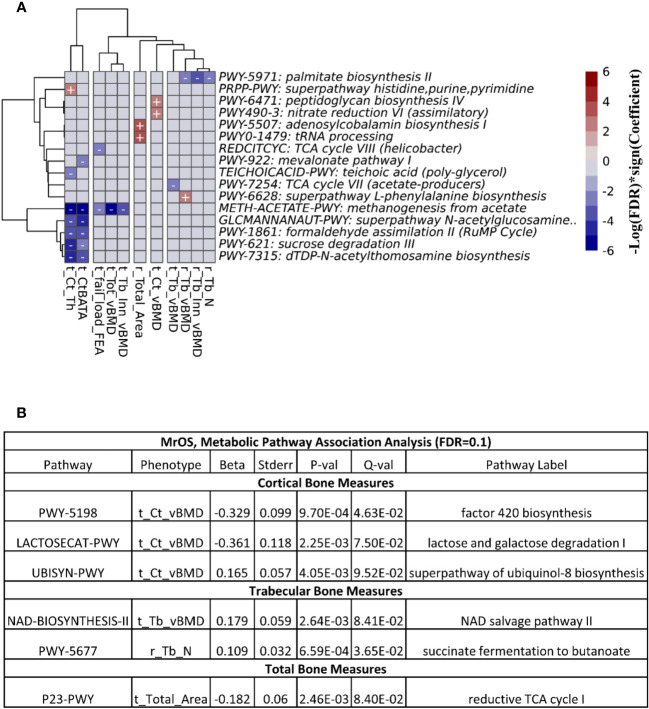
Association between predicted metabolic pathways and bone measures. The metabolic pathways were predicted using PICRUSt2. Pathways - bone associations were calculated using MaAslin2 and all associations with an FDR<0.1 are shown. We adjusted for all covariates in the association models. For **(A)** the heatmap of associations in the FHS cohort are shown. The X and Y axes were clustered hierarchically. The "+" and "-" signs indicate the direction of association between microbial abundance on the vertical axis and bone measures along the horizontal axis. **(B)** is a table of the associations in MrOS.

At FDR ≤ 0.1, we observed 26 associations between the functional pathways and bone measures in the FHS cohort (**Figure 6A**). The majority of the associations were at the cortical bone compartments followed by trabecular, total and bone strength measures. In the MrOS cohort, we uncovered only 6 pathway-bone associations largely in the tibia cortical vBMD measure (**Figure 6B**). This is consistent with the number of associations we could detect when testing for association between the actual microbial taxa abundance and bone phenotypes. **Supplementary Tables 14** and **15** contain all the pathway-bone association results up to FDR ≤ 0.25, for FHS and MrOS respectively.

Next, we meta-analyzed the pathway-bone association results from the two cohorts using the MMUPHin package (21). At FDR ≤ 0.25, we found 9 associations exclusively at the tibia cortical bone compartments involving 8 unique metabolic pathways (**Supplementary Figure 7**, **Table 16**). Greater abundance of the super pathway of histidine, purine, and pyrimidine biosynthesis (PRPP-PWY) was associated with moderate increase of the ratio of tibia cortical bone area to total area, and tibia cortical thickness. The remaining 7 pathways were only associated with tibia cortical thickness, with 4 pathways in positive direction, while 3 were inverse.

## 4 Discussion

This is the largest study to date focused on potential associations between the gut microbiome and high-resolution skeletal phenotypes in men and women across a wide age range. In addition to testing for associations between specific genera and skeletal phenotypes within each of the two large independent cohorts (FHS and MrOS) and in the cohorts combined by meta-analysis, we also assessed predicted functional pathway associations with skeletal phenotypes. We found a considerable number of taxa-bone associations, with the majority of the associations observed in the FHS cohort that was larger, and was comprised of male and female study participants who had a younger and wider age range, in contrast to the older male MrOS cohort.

In the FHS cohort, we found several 67 microbial associations with a variety of the multiple bone phenotypes in both trabecular and cortical bone compartments, as well as bone strength measures, for both the tibia and radius sites (**Figures 3**, **4**). In contrast, for the MrOS cohort, there were just 5 taxa associations with the vBMD measure in the cortical and trabecular bone compartments. These differences between cohorts may have arisen because of several factors including sample size, inclusion of women in FHS but not MrOS, and age differences. We attempted to define the contributions of these factors to the observed differences in the number of associations between cohorts. Despite having fewer males in FHS vs MrOS, there were a greater number of significant associations in the 544 male FHS participants (17 taxa-bone associations) in the FHS cohort compared with the 836 men in the MrOS cohort sample (5 taxa-bone associations). Furthermore, when we subsampled the same number of participants in the FHS (n=836) as the MrOS cohort, we still observed a greater number of taxa-bone associations (33 vs 5). This implies that the differences may not be primarily due to sample size or sex differences between cohorts but rather to other undefined differences. Since all the participants in both of the cohorts were community dwelling, it was not likely that between cohort differences were due to the types of changes reported by Claesson, et. al., who reported that older adults living in long-term care had different diets and microbiota than community dwelling adults (39). The same group also reported that within community dwelling older adults, the core microbiota of older participants was distinct from that previously established for younger adults, with a greater proportion of Bacteroides spp. and distinct abundance patterns of Clostridium groups (10). Since the MrOS cohort participants were about thirty years older than the FHS cohort, this age discrepancy might explain the differences in findings between the cohorts.

Despite the difference between the two cohorts in terms of sample size, sex and age distribution, we observed that the genera *DTU089*, a Clostridiales bacterium, was generally associated with lower bone density consistently in both of the two cohorts. Interestingly, a recent study in the MrOS cohort found *DTU089* to be significantly associated with lower protein intake (40), and we have previously demonstrated that protein intake is associated with higher BMD in the FHS (41). Our meta-analysis of the two cohorts further revealed microbial genera and species that were associated with skeletal phenotypes (**Figure 5**; **Supplementary Tables 10**, **11**). Greater abundances of *Akkermansia* and *DTU089* were associated with lower radius total vBMD, and tibia cortical vBMD respectively. Notably, a mouse study showed that treatment of gonadal-intact mice with pasteurized *Akkermansia* resulted to a reduction in trabecular and cortical bone mass (42). The findings for *DTU089* were consistent when we examined sex-specific associations within the FHS cohort as well as meta-analyzing the FHS male subgroup with MrOS, even though the sample size of these subset analyses was reduced (**Supplementary Figures 4**-**6**). Conversely, higher abundances of *Lachnospiraceae NK4A136 group*, and *Faecalibacterium* were associated with greater tibia cortical vBMD. We also investigated the functional capabilities of the microbial taxa by testing for association between predicted metabolic pathways and bone phenotypes. As expected, we observed more associations in the FHS cohort compared to MrOS, but there were no concordant associations observed in both cohorts at an FDR ≤ 0.1 (**Figure 6**). When we further meta-analyzed the two cohorts for functional associations, we found 8 metabolic pathways associated with bone measures of the tibia cortical compartment, with the most significant one being the super-pathway of histidine, purine, and pyrimidine biosynthesis (**Supplementary Figure 7**, **Table 16**). Indeed, the importance of purine metabolism in bone remodeling has been reviewed (43). Interestingly, recent mouse studies have implicated a disorder of purine metabolism to osteoporosis (44). These specific findings across the two cohorts suggest that there may be biologically based pathways linking the gut microbiome with bone metabolism. In addition, the patterns at the radius in which microbiota were negatively associated with bone density but positively association with bone area suggests the possibility that certain microbes could influence periosteal expansion at the expense of bone density, similar to what is observed with aging (45).

Our study expands on a previous analysis of the gut microbiome and skeletal phenotypes measured using HR-pQCT in the MrOS cohort alone (9). There were some notable differences in that report and the current analyses using both the MrOS and FHS cohorts. First, the work by Orwoll et al. adjusted for unique covariates that were not included in the current combined cohort analyses, such as self-rated overall health, physical activity, and a different dietary intake variable obtained using factor analysis i.e. “western” and “prudent” dietary patterns (9). There were also differences in the number and types of HR-pQCT bone phenotypes included in the analysis. For example, in the current paper we did not include cortical porosity because volumetric cortical bone density includes the contribution of cortical pores, whereas cortical porosity is limited to being able to detect only moderately large pores. In contrast with the previous MrOS publication, we did not examine dual energy x-ray absorptiometry derived phenotypes because of lack of availability of that measure in the FHS cohort at the time of stool sampling. Also, in the current report we investigated the contribution of the gut microbiota to skeletal size as reflected by the inclusion of cross-sectional area, and we investigated one additional trabecular density measure localized to the inner part of the trabecular compartment of the radius and tibia. Another important difference between our current MrOS analysis and the previously published paper on just the MrOS cohort (9), is that we separately analyzed taxa abundance at genus level and species level in contrast to the previous MrOS paper where the association analyses were restricted to the genus level. Despite these differences, at a less strict FDR ≤ 0.25, a comparison of the taxa-bone association results from the previous and current independent analyses of the MrOS cohort were concordant to a great degree. Of the 37 and 25 distinct genera detected in the previous and the current MrOS analysis, respectively, the abundance of 19 genera were observed to be associated with various bone measures in both analyses.

We were able to determine associations between the gut microbiome and skeletal measures in men and women across a wide age span using two independent cohorts. Nevertheless, several limitations should be acknowledged. Although both cohorts had the same phenotypes measured using the same generation scanners, used the same exclusion criteria, adjusted for the same covariates, and had amplicon sequencing of the V4 region of the rRNA gene, two different sequencing labs were involved, and the depth of sequencing was greater for FHS compared with MrOS, which could have resulted in differences observed between cohorts. Despite this being the largest study to date, our total sample size was still modest and associations could have been missed due to this factor.

There are many strengths in our current study. First, we addressed many of the suggested issues from a recent editorial on the microbiome and skeletal phenotypes, namely, the importance of formulating the research question and study design, sequencing technology, and data analytical methods (46). We investigated microbiota – bone associations using 16S rRNA amplicon sequencing in a larger sample of men and women with a wider age range who had state-of-the-art measures of bone density, microarchitecture and strength derived from HR-pQCT imaging of the radius and tibia. We also employed methods to examine genera level associations and where possible, species level associations with bone measures. We explored pathway analyses using data from both cohorts. Although there were distinct differences in findings between the two cohorts, there were several consistent findings that we were able to replicate between cohorts. Another strength of this study is that both FHS and MrOS are extensively phenotyped cohorts, thus providing us with sufficient information about study participant’s clinical characteristics. Finally, there were several findings with biologic plausibility based on animal studies of specific bacterial species.

In conclusion, our findings of several consistent associations between gut microbiota and skeletal measures suggest that there is likely to be a link between the gut microbiome and skeletal metabolism. With larger samples and future availability of shotgun sequencing, we might be able to gain additional insights regarding the specific species that influence the skeleton. This may lead to targeting of the gut microbiome to influence skeletal health.

## Data availability statement

The datasets presented in this study can be found in online repositories. The names of the repository/repositories and accession number(s) can be found below: https://www.ncbi.nlm.nih.gov/gap/, pht006014.v3.p14. https://mrosonline.ucsf.edu/, Codes used in this analysis can be found at https://github.com/hebrewseniorlife/FHS-MrOS-16S, https://www.ncbi.nlm.nih.gov/, PRJNA758252, https://www.ebi.ac.uk/ena, PRJEB26012.

## Ethics statement

The studies involving humans were approved by Advarra Institutional Review Board. The studies were conducted in accordance with the local legislation and institutional requirements. The participants provided their written informed consent to participate in this study.

## Author contributions

PCO performed the analyses, drafted the manuscript, and contributed to the interpretation of results. ESO and DPK contributed to data collection, study conceptualization, drafting of the analysis plan, data curation, formal analysis, interpretation of results, reviewing of manuscript, and supervision of the study. SS contributed to analysis, interpretation of results, and reviewing of the manuscript. CH contributed to data curation, interpretation of results, and reviewing of the manuscript. XM and TMK contributed to data curation, analysis, interpretation of results and reviewing of the manuscript. RP contributed to interpretation of results and reviewing of manuscript. MLB contributed to data curation, and interpretation of results. DMK contributed to the MrOS data collection and reviewing of the manuscript. LJM contributed to data curation, and analysis. ABD contributed to data curation, analysis, and interpretation of results. LS and SF contributed in reviewing of the manuscript. All authors contributed to the article and approved the submitted version.
