# Supplementary material for: A two-cohort study on the association between the gut microbiota and bone density, microarchitecture, and strength
Source: Front Endocrinol (Lausanne). 2023 Sep 21;14:1237727. doi: 10.3389/fendo.2023.1237727 (PMC10551180; doi:10.3389/fendo.2023.1237727)
Supplement: Supplementary file 1 [file DataSheet_1.pdf]

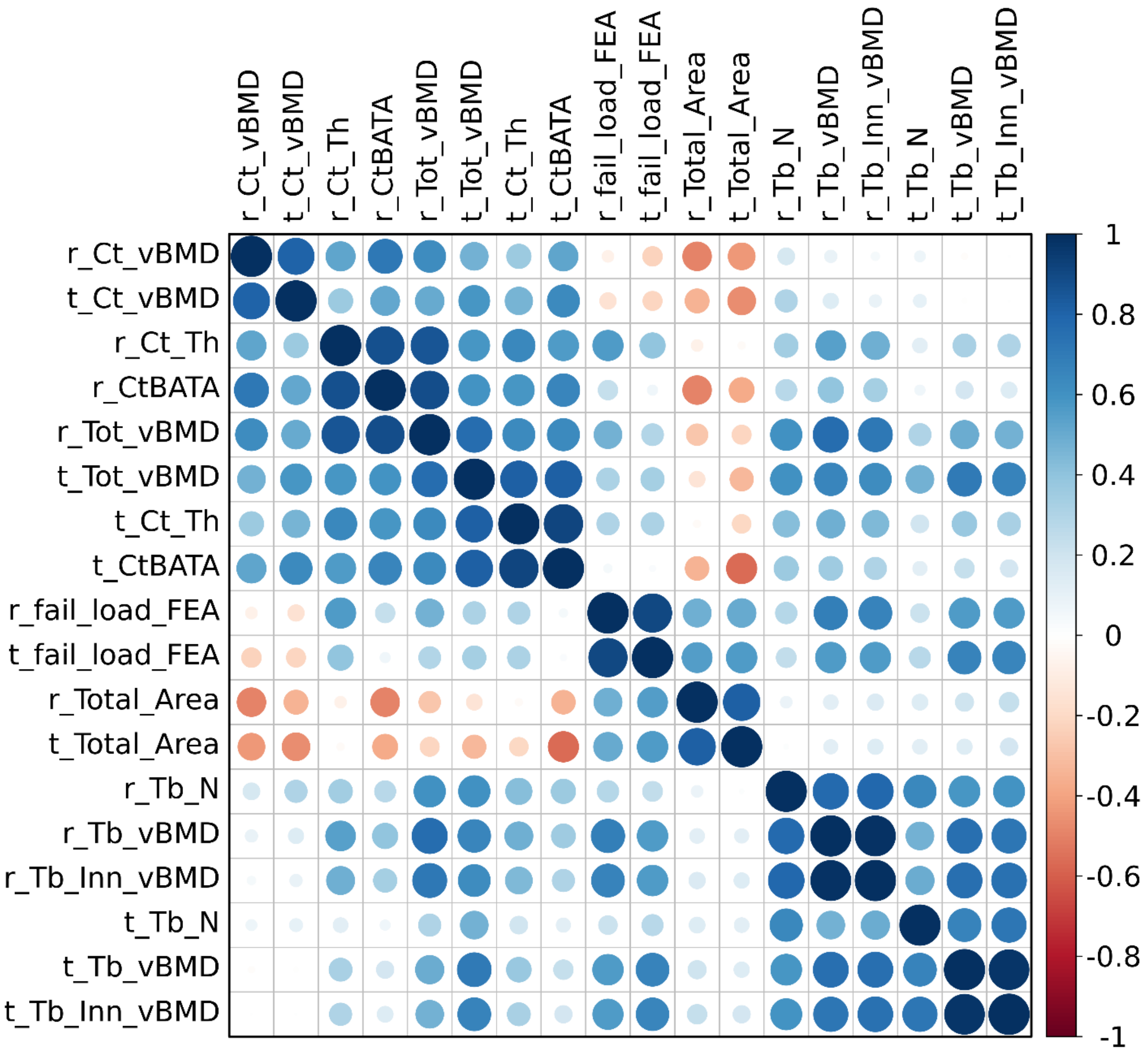

S Figure 1. Correlation heatmap of the High Resolution Peripheral Quantitative Computed Tomography (HR-pQCT) bone measures. Highly correlated measures were grouped using hierarchical clustering.

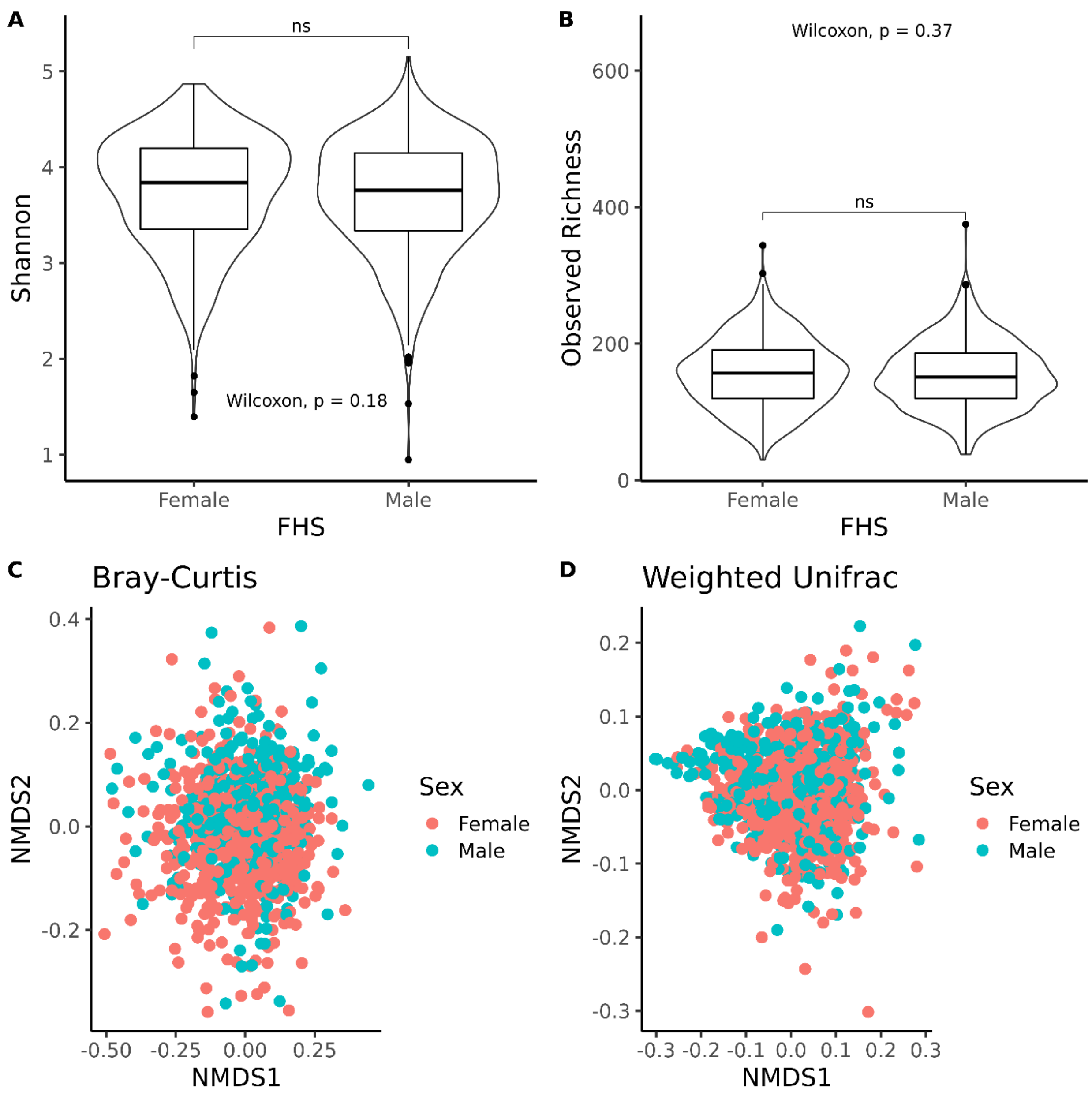

S Figure 2. Comparison of the microbial diversity of the male and female participants in the FHS cohort. Microbial taxa were glomed at genus level. Panel A and B shows the alpha diversity; Shannon and observed genera (richness) respectively, where "ns" means not significant between the two groups. Specifically, for Panel B, samples were rarefied before estimating richness. Panel C and D shows the beta diversity estimated using Bray Curtis distance (stress=0.20) and weighted UNIFRAC distance (stress=0.16) respectively, and visualized using the Non-Metric Multidimensional Scaling (NMDS). Panel C PERMANOVA analysis is R^2=0.005, F=5.933, P=0.001, while for panel D is R^2=0.009, F=11.173, P=0.001. We adjusted for all covariates in the PERMANOVA tests.

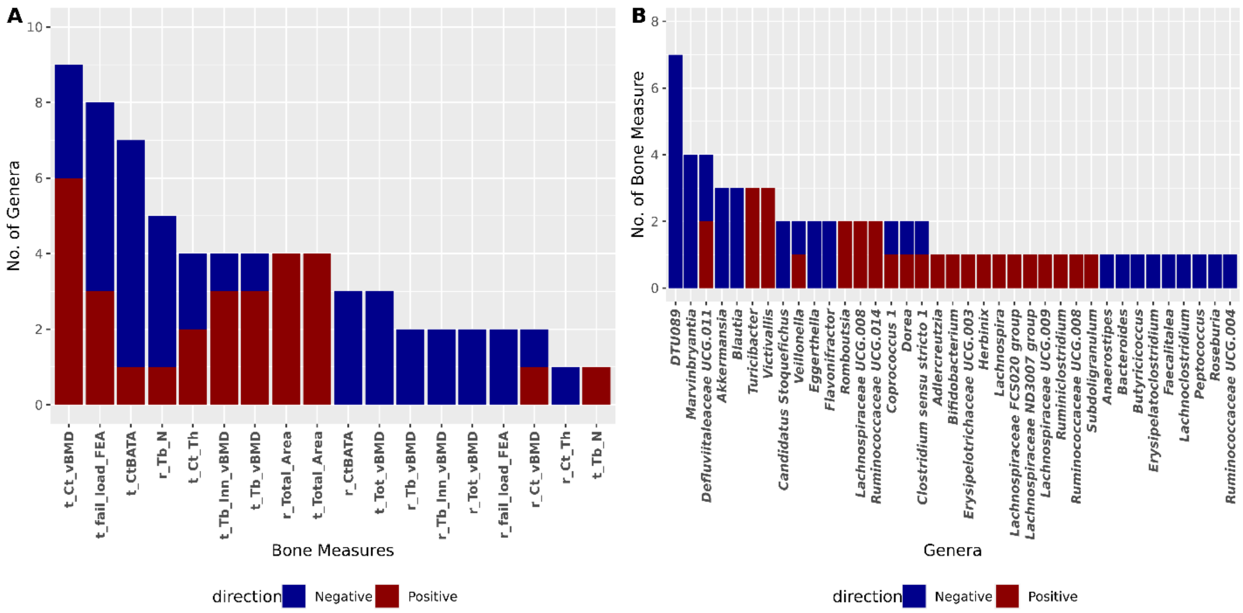

S Figure 3. Distribution of the observed associations between microbial genera and bone measures in FHS. Panel A displays the total numbers of microbial genera associated with each bone measure at an FDR ≤0.1 in the FHS cohort. In Panel B, the total number of bone measures associated with bacterial genera are shown at an FDR ≤0.1 Positive directions of association are indicated by the red color and negative directions of association by the blue color.

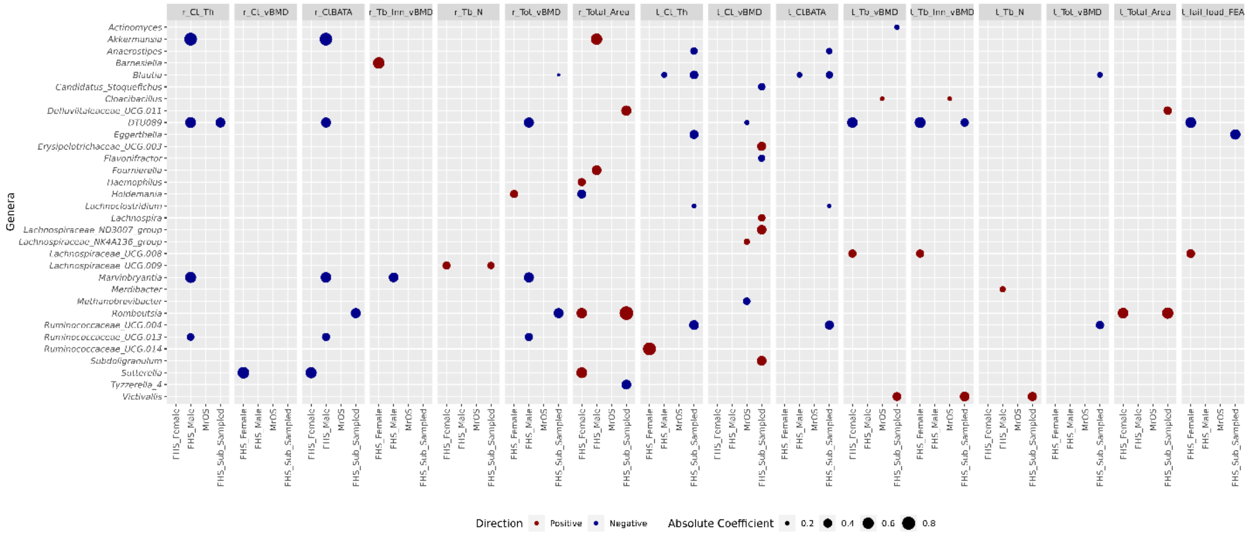

S Figure 4. Associations between microbial genera and bone measures at FDR ≤ 0.1 in the FHS sex specific and subsampled cohort, and in the MrOS cohort. The horizontal axis provides the specific cohort(s) and sex used in the analysis. The vertical axis displays the genera associated with the bone measures indicated by the vertical panels. Dot size reflects the magnitude of association with blue color indicating a negative association between abundance and a bone measure while a red color indicates a positive association. We adjusted for all covariates in the association models.

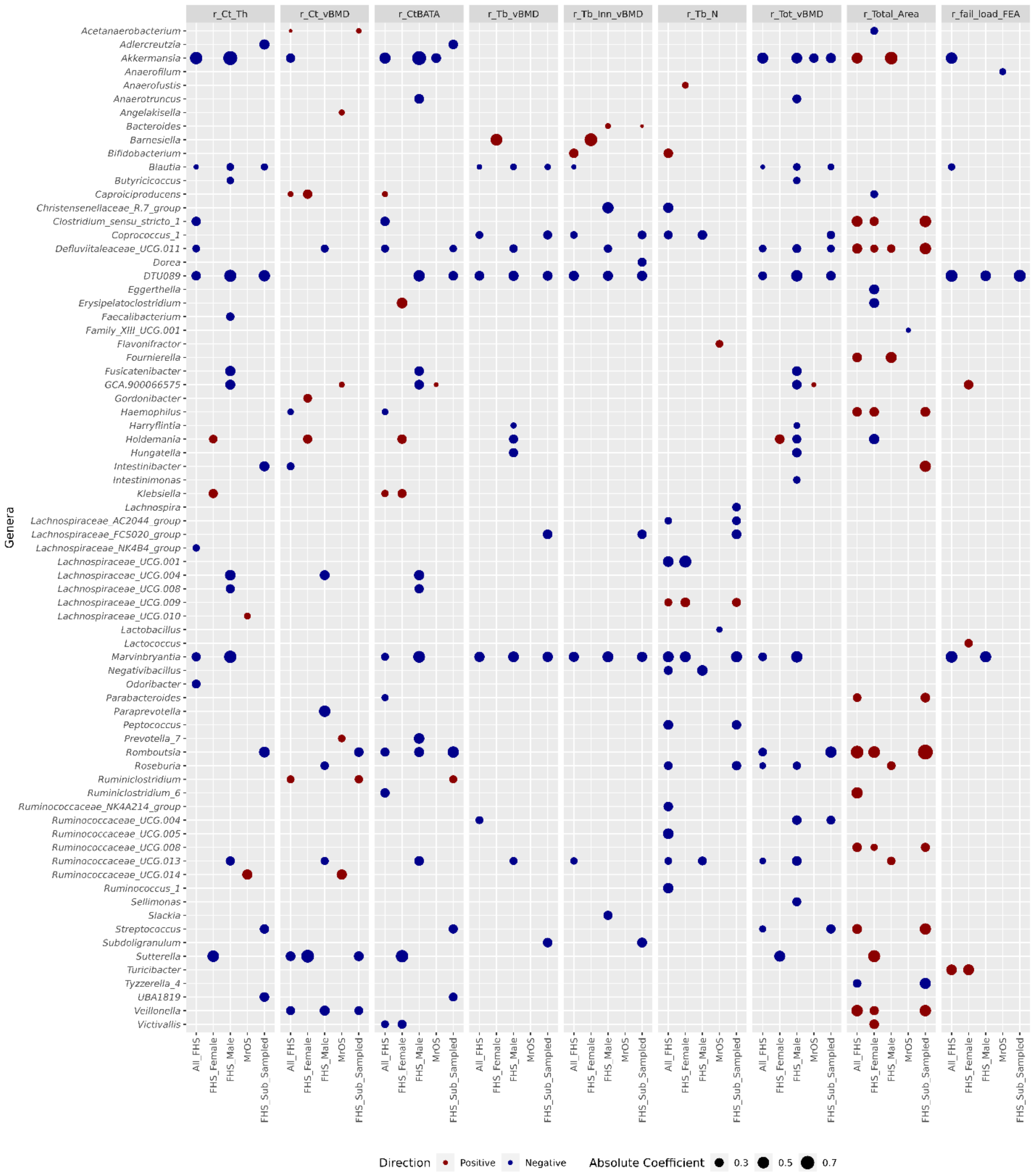

S Figure 5. All associations between microbial genera and distal radius bone measures at FDR ≤ 0.25 in the FHS, FHS-sex specific and subsampled cohorts, and in the MrOS cohort. The horizontal axis provides the specific cohort(s) and sex used in the analysis. The vertical axis displays the genera associated with the bone measures indicated by the vertical panels. Dot size reflects the magnitude of association with blue color indicating a negative association between abundance and a bone measure while a red color indicates a positive association. We adjusted for all covariates in the association models.

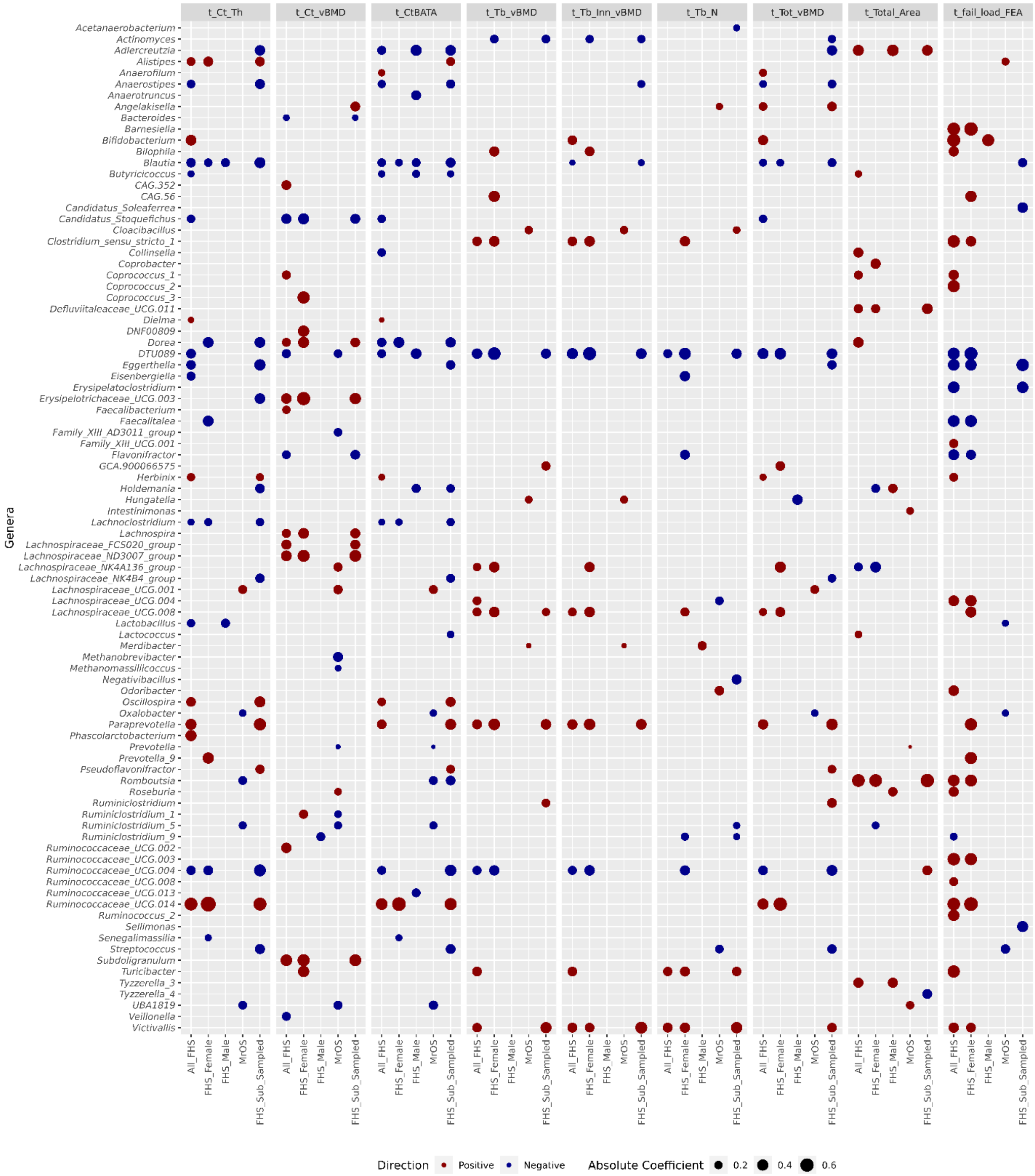

S Figure 6. All associations between microbial genera and distal tibia bone measures at FDR ≤ 0.25 in the FHS, FHS-sex specific and subsampled cohorts, and in the MrOS cohort. The horizontal axis provides the specific cohort(s) and sex used in the analysis. The vertical axis displays the genera associated with the bone measures indicated by the vertical panels. Dot size reflects the magnitude of association with blue color indicating a negative association between

abundance and a bone measure while a red color indicates a positive association. We adjusted for all covariates in the association models.

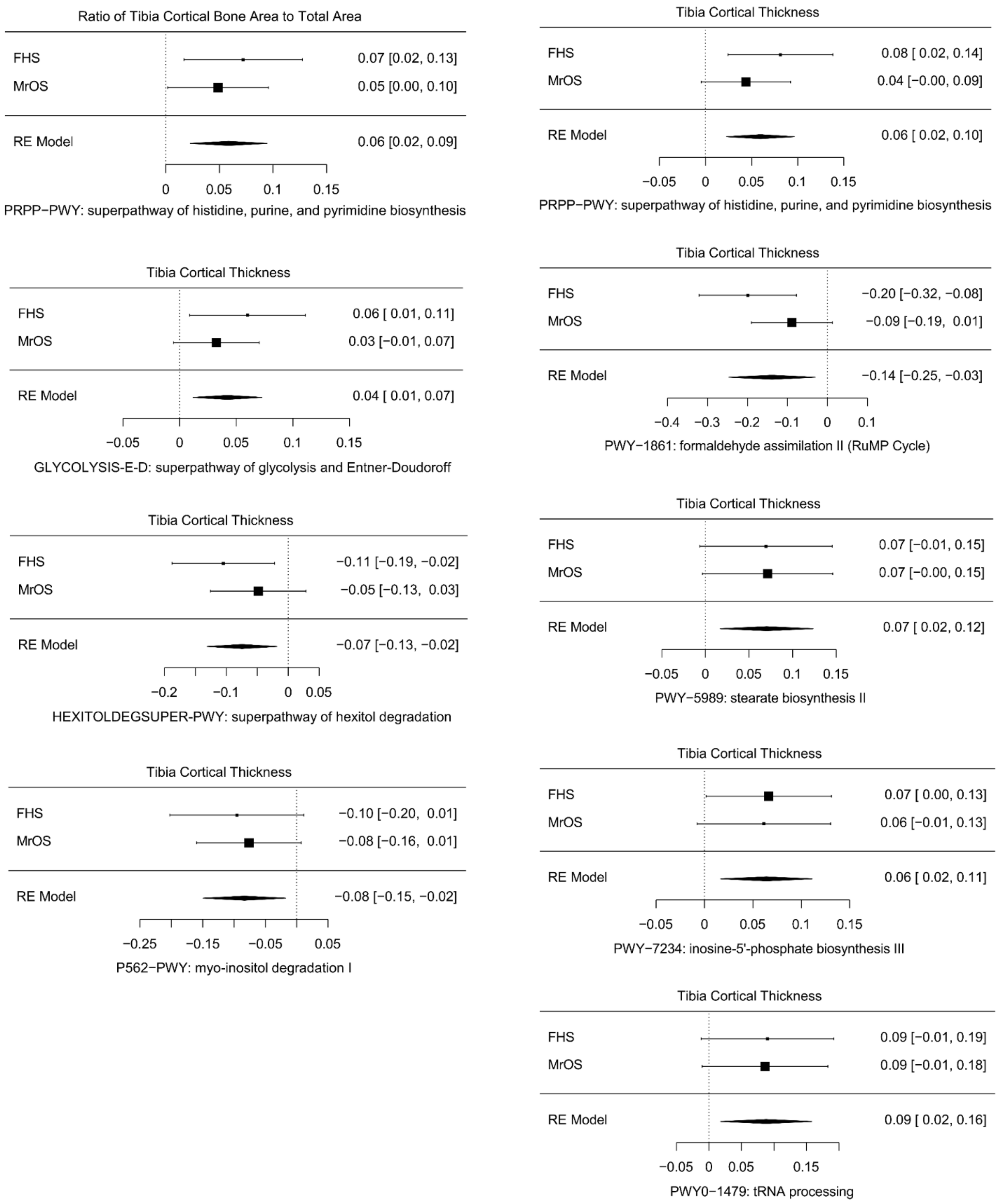

S Figure 7. Meta-analysis of the associations between abundance of predicted metabolic pathway and bone measures. Meta-analysis of pathways – bone associations was conducted with MMUPHin and all associations at FDR ≤ 0.25 are shown.
